# Compound heterozygous splice site variants in the *SCLT1* gene highlight an additional candidate locus for Senior-Løken syndrome

**DOI:** 10.1038/s41598-018-35152-6

**Published:** 2018-11-13

**Authors:** Satoshi Katagiri, Takaaki Hayashi, Kazutoshi Yoshitake, Noriyuki Murai, Zenichi Matsui, Hiroyuki Kubo, Hiroyuki Satoh, Senya Matsufuji, Tsuyoshi Takamura, Takashi Yokoo, Yoshihiro Omori, Takahisa Furukawa, Takeshi Iwata, Tadashi Nakano

**Affiliations:** 1 Department of Ophthalmology, The Jikei University School of Medicine, Tokyo, Japan; 2 Department of Ophthalmology, Katsushika Medical Center, The Jikei University School of Medicine, Tokyo, Japan; 3 Division of Molecular and Cellular Biology, National Institute of Sensory Organs, Tokyo Medical Center, National Hospital Organization, Tokyo, Japan; 4 Department of Molecular Biology, The Jikei University School of Medicine, Tokyo, Japan; 5 Department of Urology and Kidney transplants, Tokyo Metropolitan Children’s Medical Center, Tokyo, Japan; 6 Department of Ophthalmology, Kanagawa Rehabilitation Hospital, Kanagawa, Japan; 7 Division of Nephrology and Hypertension, Department of Internal Medicine, The Jikei University School of Medicine, Tokyo, Japan; 8 Laboratory for Molecular and Developmental Biology, Institute for Protein Research, Osaka University, Osaka, Japan

## Abstract

Senior Løken syndrome (SLS) is a heterogeneous disorder characterized by severe retinal degenerations and juvenile-onset nephronophthisis. Genetic variants in ten different genes have been reported as the causes of SLS. Clinical evaluation of a patient with SLS and her unaffected parents revealed that the patient had infantile-onset retinal dystrophy and juvenile-onset nephronophthisis. Other systemic abnormalities included hepatic dysfunction, megacystis, mild learning disability, autism, obesity, and hyperinsulinemia. Whole-exome sequencing identified compound heterozygous *SCLT1* variants (c.1218 + 3insT and c.1631A > G) in the patient. The unaffected parents were heterozygous for each variant. Transcript analysis using reverse transcription PCR demonstrated that the c.1218 + 3insT variant leads to exon 14 skipping (p.V383_M406del), while the other variant (c.1631A > G) primarily leads to exon 17 skipping (p.D480EfsX11) as well as minor amounts of two transcripts (6 bps deletion in the last of exon 17 [p.V543_K544del] and exons 17 and 18 skipping [p.D480E, S481_K610del]). Immunohistochemical analysis demonstrated that the Sclt1 protein was localized to the distal appendage of the photoreceptor basal body, indicating a ciliary protein. In conclusion, we identified compound heterozygous splice site variants of *SCLT1* in a patient with a new form of ciliopathies that exhibits clinical features of SLS.

## Introduction

Senior Løken syndrome (SLS) is a heterogeneous disorder characterized by severe retinal degenerations and juvenile-onset nephronophthisis (NPHP)^1^. SLS was first described in the literature by Senior *et al*.^2^ and Løken *et al*.^3^ in 1961. Retinal degenerations show broad phenotypes from Leber congenital amaurosis to mild forms of retinitis pigmentosa^1,4^. NPHP is a medullary cystic kidney disease that can lead to end-stage renal disease in both children and adolescents^5^.

Genetic variants in the *NPHP1* gene were first identified as being the causes of SLS in 1997^6^. Subsequently, at least variants in ten genes have been reported to cause SLS, including the *NPHP1*^6,7^, *NPHP2*^8^, *NPHP3*^9^, *NPHP4*^10^, *NPHP5/IQCB1*^11^, *NPHP6/CEP290*^12,13^, *NPHP10/SDCCAG8*^14^, *NPHP13/WRD19*^15,16^, *NPHP15/CEP164*^17^ and *TRAF3IP1*^18^ genes. The proteins produced by these genes play a pivotal role in cellular structures called the cilium, which is found in most cell types. For example, they are involved with the cilia connecting the inner to the outer segments in both the rod and cone photoreceptors, the cilia in the sensory hair cells of the inner ear, and the cilia in the renal tubular epithelial cells of the kidney^5,19^. Disruption of the ciliary structure and function leads to multiple organ diseases, which are referred to as ciliopathies, with SLS considered to be one form of these ciliopathies^5,19^.

In the current study, we determined that the compound heterozygous splice site variants in the sodium channel and clathrin linker 1 *(SCLT1*) gene were the cause of SLS in a Japanese patient. This is the first report to show that the *SCLT1* variants are the cause of SLS. Therefore, the purpose of this study was to report the clinical and genetic features of this SLS patient and the cellular localization of the Sclt1 protein in the retina.

## Results

### Clinical presentation

**Figure 1:**
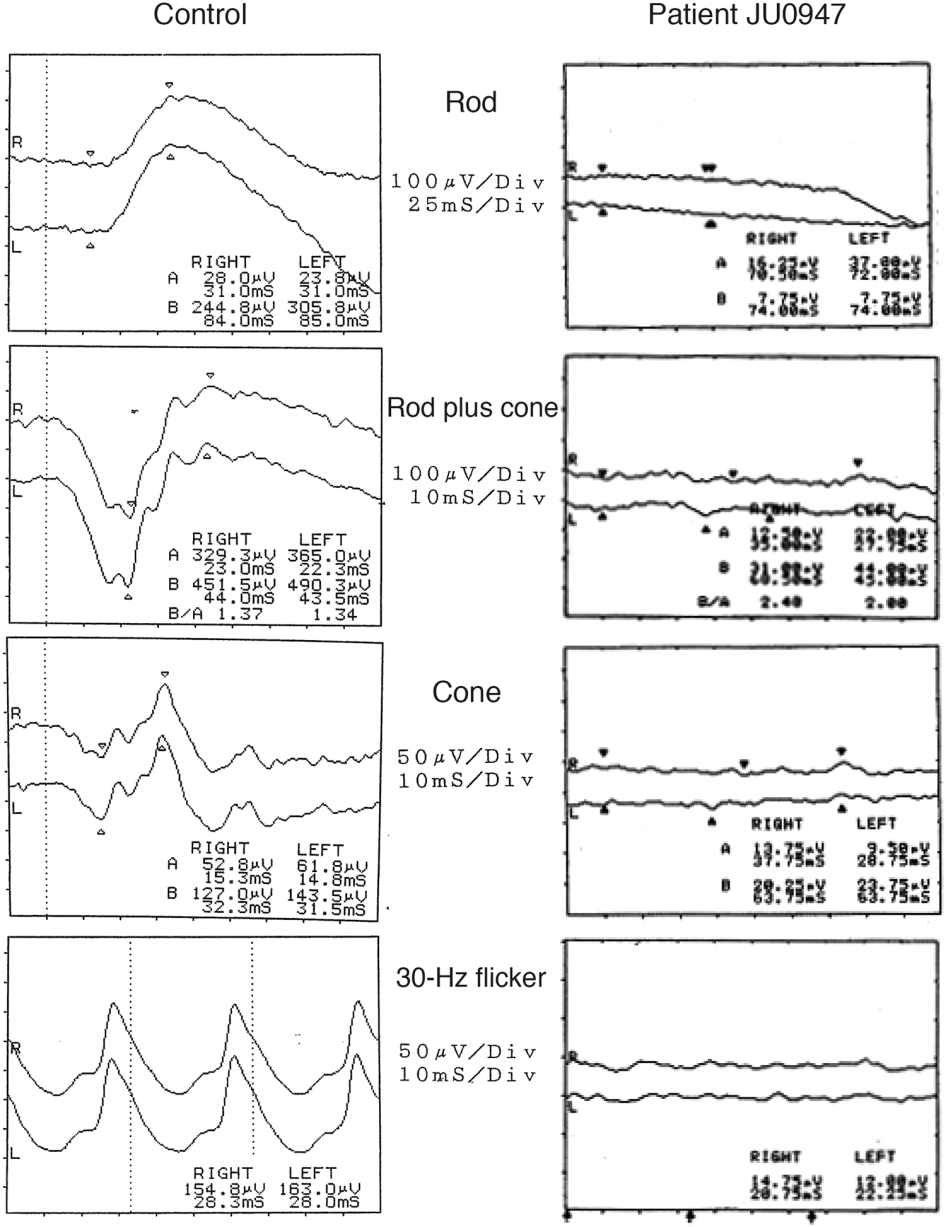
Patient full-field electroretinography results. Full-field electroretinography at the age of 6 years shows non-recordable patterns in the rod, combined rod plus cone, cone and 30-Hz flicker responses.

**Figure 2:**
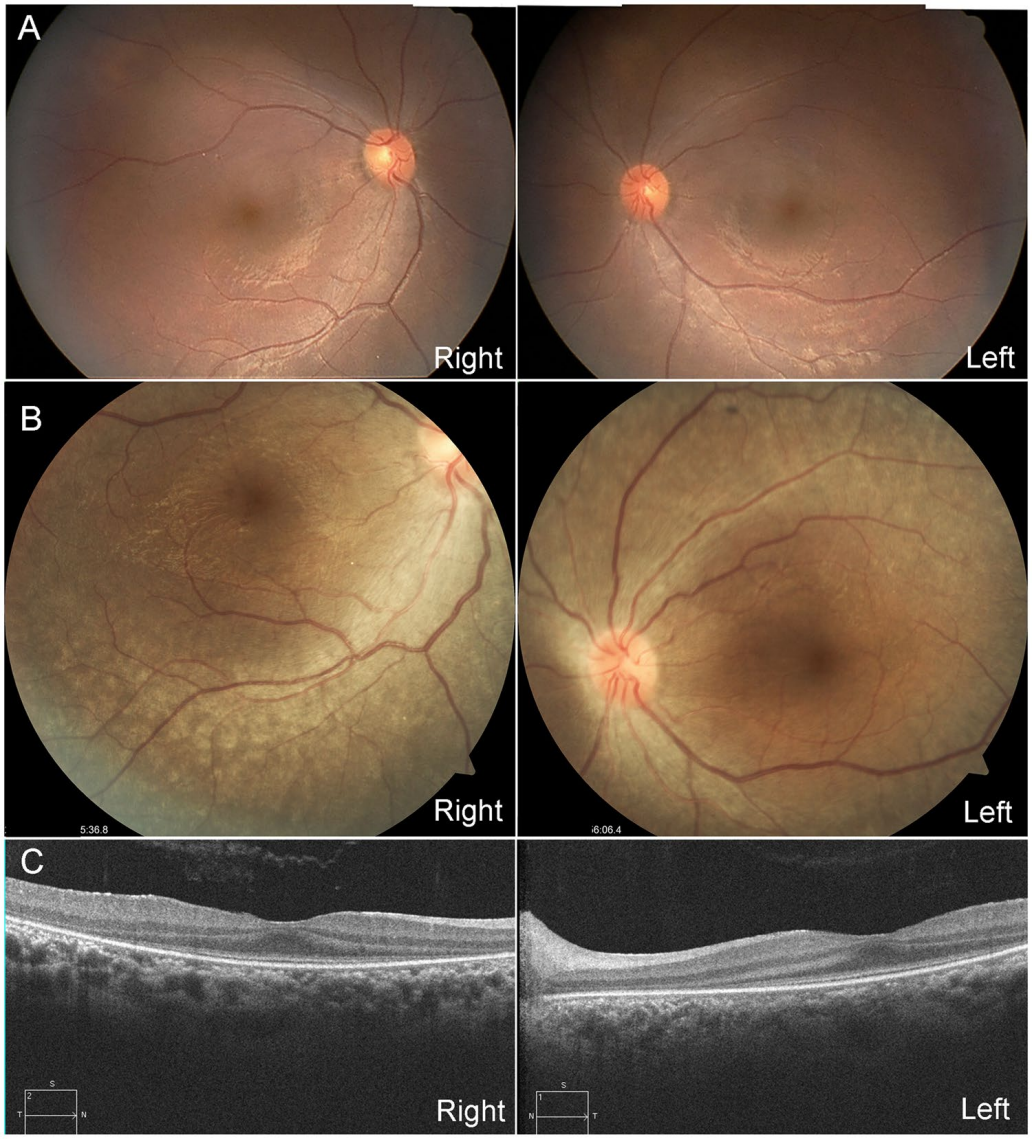
Patient fundus photographs and optical coherence tomography results. (**A**) Fundus photographs at the age of 6 years show no remarkable findings in either of the patient’s eyes. (**B**) Fundus photographs at the age of 13 years show retinal degenerations ranging from the vascular arcades to the mid-periphery in both eyes. **(C**) Spectral-domain optical coherence tomography at the age of 13 years show thinning of the outer retinal layers with a blurred ellipsoid zone in the foveal and parafoveal regions, and the disappearance of the ellipsoid zone outside of these regions in both eyes.

**Figure 3:**
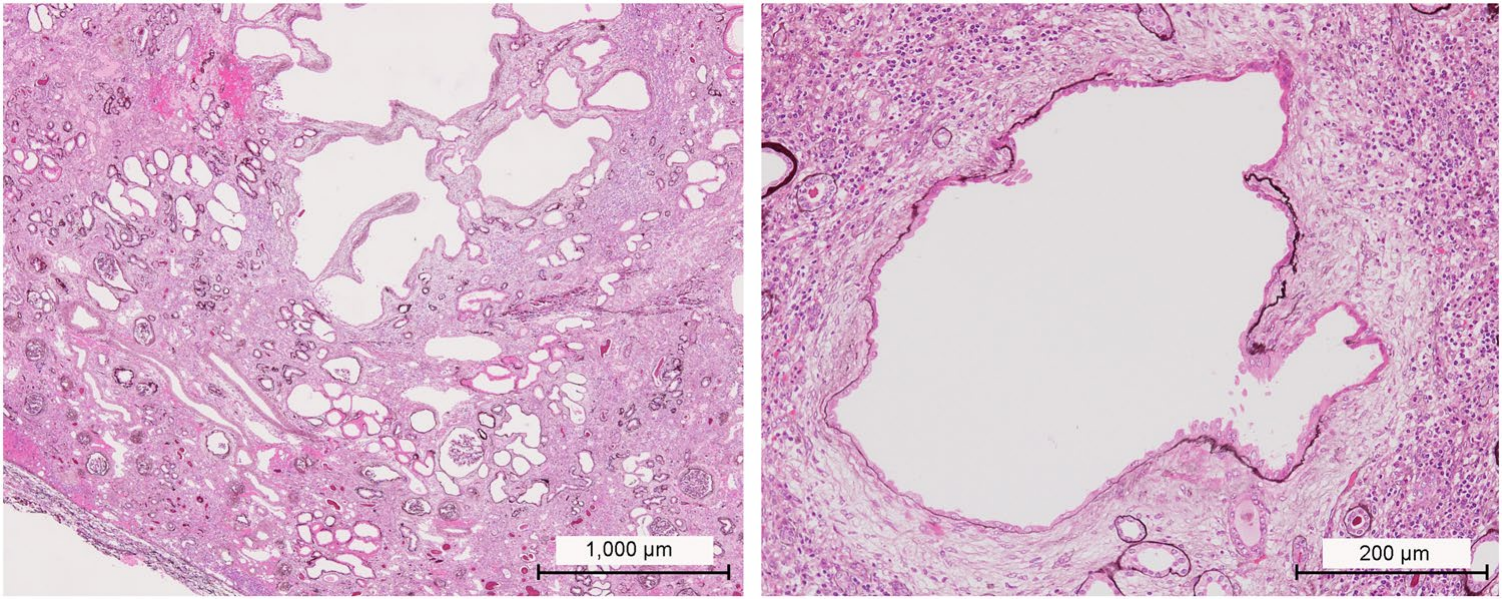
Histological examinations of the nephrected kidney of the patient. Histological examinations of the nephrected kidney at the age of 12 years. Object lens x4 (**A**) and 20 (**B**) with periodic acid methenamine silver staining. Numerous tubular cysts, disruption of the tubular basement membranes, and interstitial infiltration with interstitial fibrosis are seen in the images.

**Figure 4:**
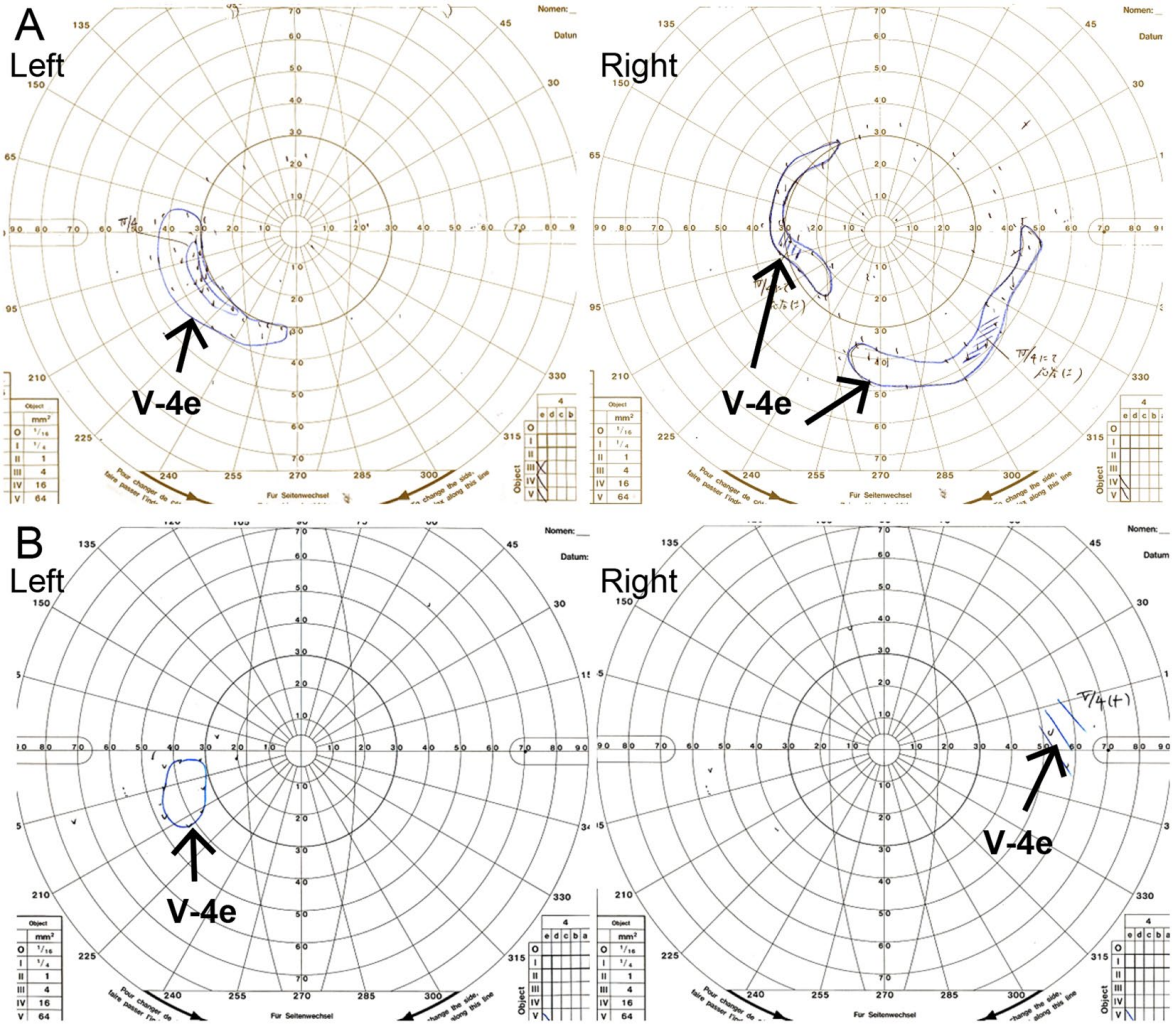
Patient visual field testing. (**A**) Kinetic visual field testing at the age of 13 years shows small residual mid-peripheral visual fields in both eyes. (**B**) Kinetic visual field testing at the age of 18 years shows only a small island in the periphery of both eyes.

**Figure 5:**
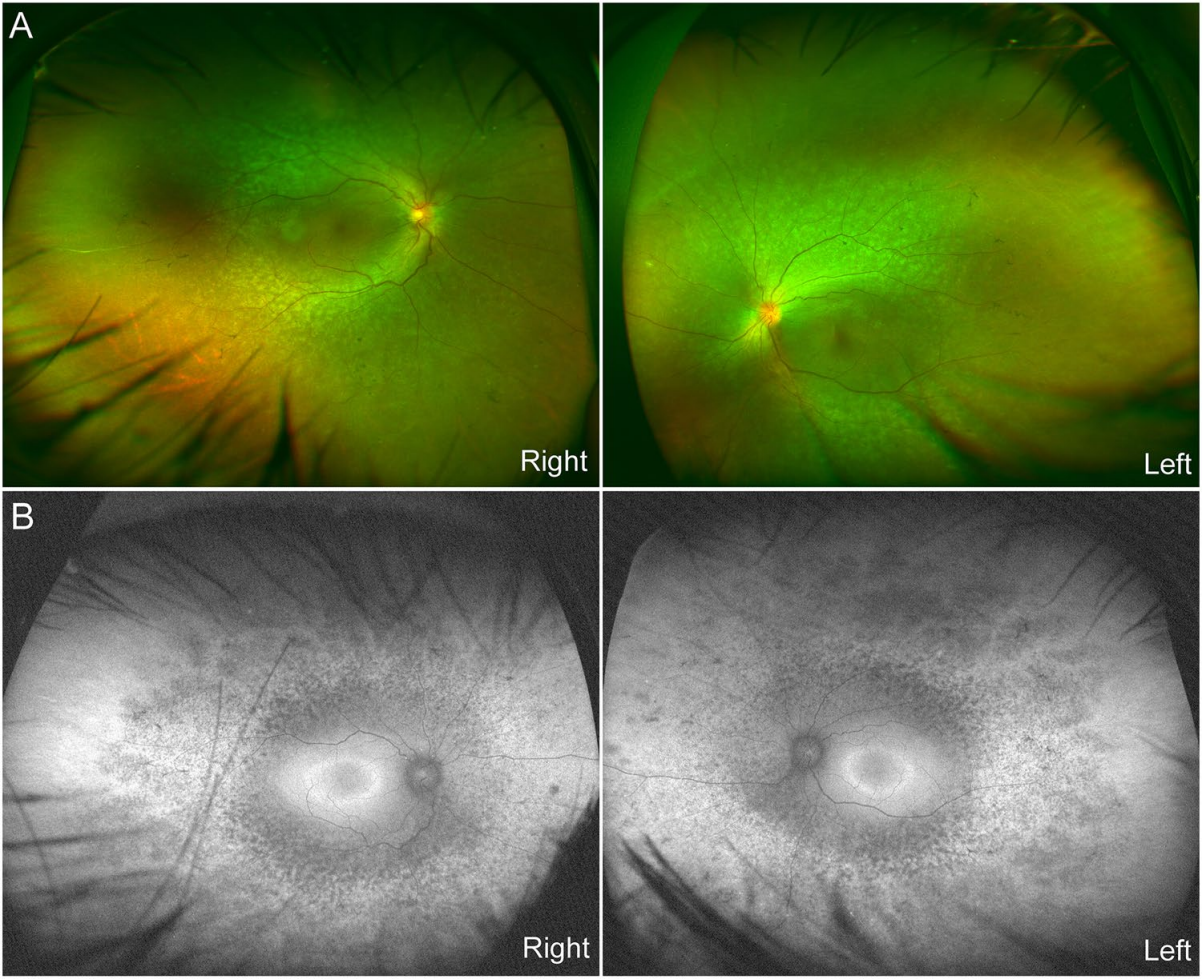
Patient wide-field fundus photographs and fundus autofluorescence imaging. (**A**) Wide-field fundus photographs show retinal degeneration from the vascular arcade to the mid-periphery, along with attenuated retinal vessels in both of her eyes. (**B**) Fundus autofluorescence images show mottled hyperautofluorescence and hypoautofluorescence that corresponds to the retinal degeneration, and a hyperfluorescent ring around the macula in both eyes.

A 1-year-old female patient (JU0947, II-2) was referred to her previous doctor because of poor visual fixation. She had nystagmus and photophobia at that time, but did not have midline cleft lip and palate, microcephaly and choanal atresia. There was no parental consanguinity. At 6 years of age, the patient was ophthalmologically examined because of a loss of her vision. Decimal best-corrected visual acuity (BCVA) was 0.03 (with +7.25 diopters) in both eyes. High exotropia and nystagmus were observed. Full-field electroretinography showed non-recordable patterns in the rod, combined (rod plus cone), cone, and 30-hertz flicker responses (Fig. 1). The electroretinographic findings demonstrated severe retinal dysfunction. However, there were no remarkable findings noted during funduscopy (Fig. 2A). The patient was diagnosed as having either early-onset severe retinal dystrophy or Leber congenital amaurosis. At the age of 10 years, the patient was subsequently found to have urinary sugar. Systemic examinations disclosed multi-organ disorders, including juvenile NPHP, hepatic dysfunction, megacystis, mild learning disability, autism, obesity, and hyperinsulinemia. Peritoneal dialysis was started at the age of 11 years. Due to renal hypertension and dysfunction, the patient underwent nephrectomy of both kidneys followed by kidney transplantation at the age of 12 years. Histopathological examinations of the nephrected kidney showed numerous tubular cysts, disruption of tubular basement membranes, and interstitial infiltration with interstitial fibrosis (Fig. 3), which indicated NPHP. At the age of 13 years, BCVA decreased to 0.01 in both eyes. Funduscopy showed retinal degenerations from the vascular arcades to the mid-periphery in both eyes (Fig. 2B). Spectral-domain optical coherence tomography (OCT) showed thinning of the outer retinal layers with a blurred photoreceptor ellipsoid zone (EZ) in the foveal and parafoveal regions and disappearance of EZ outside of these regions in both eyes (Fig. 2C). Goldmann perimetry (GP) revealed small residual mid-peripheral visual fields in both eyes (Fig. 4A). At the age of 18 years, decimal BCVA still remained at 0.01 in both eyes. GP showed only a small island in the periphery of each eye (Fig. 4B). Wide-field fundus photographs demonstrated that there were retinal degeneration from the vascular arcade to the mid-periphery, and attenuated retinal vessels (Fig. 5A). Fundus autofluorescence images revealed mottled hyperautofluorescence and hypoautofluorescence that corresponded to the retinal degeneration, along with a hyperfluorescent ring around the macula in both eyes (Fig. 5B).

### Genetic analysis

**Figure 6:**
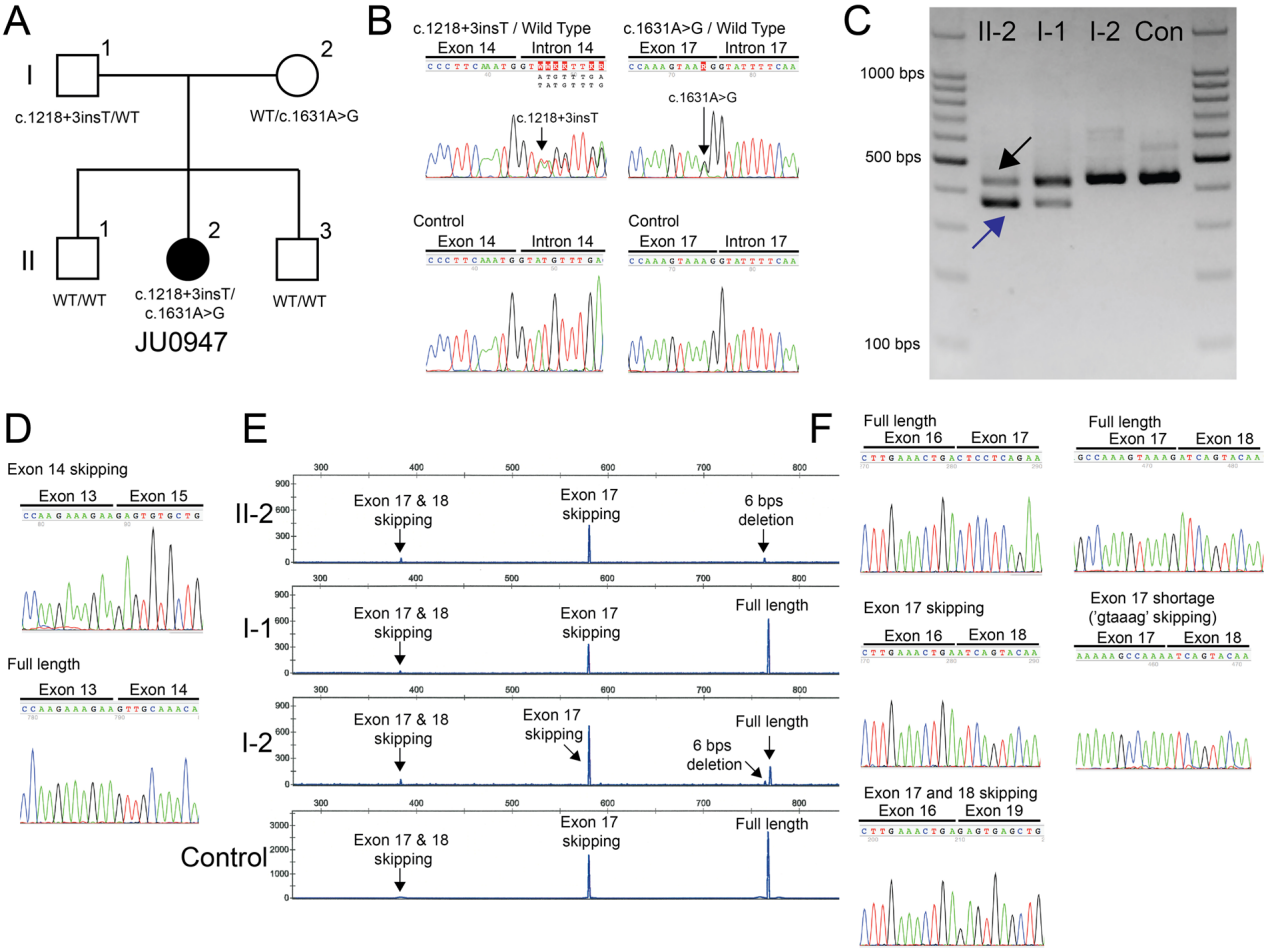
Pedigree of the Japanese family, identified *SCLT1* variants and transcript analysis. (**A**) Patient (female, solid circle) and unaffected family members (males, open squares; female, open circle) are shown. (**B**) Partial sequence data of the compound heterozygous *SCLT1* variants (c.1218 + 3insT and 1631A > G). (**C**) Agarose gel electrophoresis of the reverse transcription-PCR product using the primer pair of SCTL1-E13F and SCLT1-E17R. The patient (II-2) and father (I-1) show two bands whereas the mother (I-2) and control (Con) show a single band. (**D**) Partial sequence data of the cloning product using the primer pair of SCTL1-E13F and SCLT1-E17R. For instance, the sequence of the shorter band (blue arrow in C) reveals exon 14 skipping while that for the longer band (black arrow in C) shows the full-length product. (**E**) The results of GeneMapper using the reverse transcription-PCR product with a primer pair of SCLT1-E14F and FAM labeled SCLT1-E20R. Each sample shows different patterns and four distinct peaks, which were approximately 768, 763, 580, and 383 bps. (**F**) Partial sequence data of cloning product using the primer pair of SCTL1-E14F and SCLT1-E20R. The product is around 768 bps, the full length of the SCLT1 mRNA transcript is around 763 bps with the 6 bps deletion of the last part of Exon 17, while Exon 17 skipping is around 580 bps, and Exon 17 and Exon 18 skipping is around 382 bps.

Whole-exome sequencing was performed for the proband and his unaffected parents (Fig. 6A). The average length of the sequence was 8.6 giga base pairs. After eliminating reads with duplicated start sites, we achieved a 68-fold average depth and a 93% coverage for at least 5 depths in the RefSeq-annotated regions. After the filtering steps, there were 5 variants in 3 genes that remained (Supplementary Table S1). Among these genes, compound heterozygous *SCLT1* variants (c.1218 + 3insT in the 3 bp downstream of the exon 14-intron 14 boundary and c.1631A > G at the 2 bp upstream of the exon 17-intron 17 boundary) remained as a strong candidate, as pathogenic variants of *SCLT1* have been previously reported to be a cause of Oro-Facio-Digital syndrome type IX^20^ and non-syndromic retinitis pigmentosa (RP)^21^. Co-segregation analysis revealed that the unaffected father (I-1) had c.1218 + 3insT heterozygously while the unaffected mother (I-2) had c.1631A > G heterozygously (Fig. 6A,B). The c.1218 + 3insT has not been reported in any previous literature or databases. However, the c.1631A > G variant has been reported to have a very low frequency (Supplementary Table S1) with an uncertain significance. The other 3 variants (Supplementary Table S1) were consequently excluded by genotype-phenotype correlation analysis. No pathogenic variants were detected in the *NPHP1*^6,7^, *NPHP2*^8^, *NPHP3*^9^, *NPHP4*^10^, *NPHP5/IQCB1*^11^, *NPHP6/CEP290*^12,13^, *NPHP10/SDCCAG8*^14^, *NPHP13/WRD19*^15,16^, *NPHP15/CEP164*^17^ and *TRAF3IP1*^18^ genes, variants of which are known to be cause SLS.

### SCLT1 mRNA analysis

Reverse transcription polymerase chain reaction (RT-PCR) using SCLT1-E13F and SCLT1-E17R detected a single 442-bp band in the mother (I-2) and in the control, whereas a smaller band in addition to the 442-bp band were detected in the patient (II-2) and the father (I-1), who exhibited c.1218 + 3insT in the agarose gel (Fig. 6C). Nucleotide sequencing of the clone from each band confirmed that the 442-bp band was derived from full-length transcripts (exons 13 to 17), whereas the shorter band was derived from transcripts with exon 14 skipping, resulting in a 72-bp in-frame deletion lacking 24 amino acids (p.V383_M406del) (Fig. 6D). Regarding the c.1631A > G variant (Fig. 6B), it was a possibility that the variant leads to a missense variant (p.K544R) other than a splice site variant. Therefore we investigated the impact of the variant on splicing. For the RT-PCR for the allele with c.1631A > G, we chose SCLT1-E14F and SCLT1-E20R as primers for the procedure, as the transcripts from c.1218 + 3insT were never amplified due to exon 14 skipping in the patient. Gel electrophoresis demonstrated that the RT-PCR products had different size patterns. The GeneScan analysis of the RT-PCR products from 40 cycles showed different peak patterns among the samples (II-2, I-1, I-2, control) (Fig. 6E). Four different lengths of the products were identified, with approximately 768, 763, 580, and 383 bp, respectively. The similar GeneScan results were obtained from 35 cycles (Supplementary Fig. S1), although the dye signal intensity was weaker than that from 40 cycles. Based on the nucleotide sequences of the clones from the RT-PCR products, we confirmed that the 768-bp product was from the full-length transcripts (exons 14 to 20), while the 763-bp product was from the nearly full-length transcripts with the 6-bp deletion (at the end of exon 17), the 580-bp product was from transcripts with exon 17 skipping, and that the 383-bp product was from transcripts with the skipping of both exons 17 and 18 (Fig. 6F). The proband results reflected the mRNA transcripts of only the allele with c.1631A > G variant, as the other allele with the c.1218 + 3insT variant was not amplified by SCLT1-E14F and SCLT1-E20R due to the exon 14 skipping (Fig. 6C,D). In summary, the c.1631A > G variant resulted in primarily the mRNA transcript of exon 17 skipping (p.D480EfsX11) with minor amounts of two transcripts, the 6 bps deletion of the last part of exon 17 (p.V543_K544del) and that of exon 17 and exon 18 skipping (p.D480E, S481_K610del). Even use of the wild type primarily resulted in the mRNA transcript of full length, followed by 17 skipping, with a slight amount of exon 17 and exon 18 skipping.

### Localization of Sclt1 protein in the mouse retinal and kidney tissues

**Figure 7:**
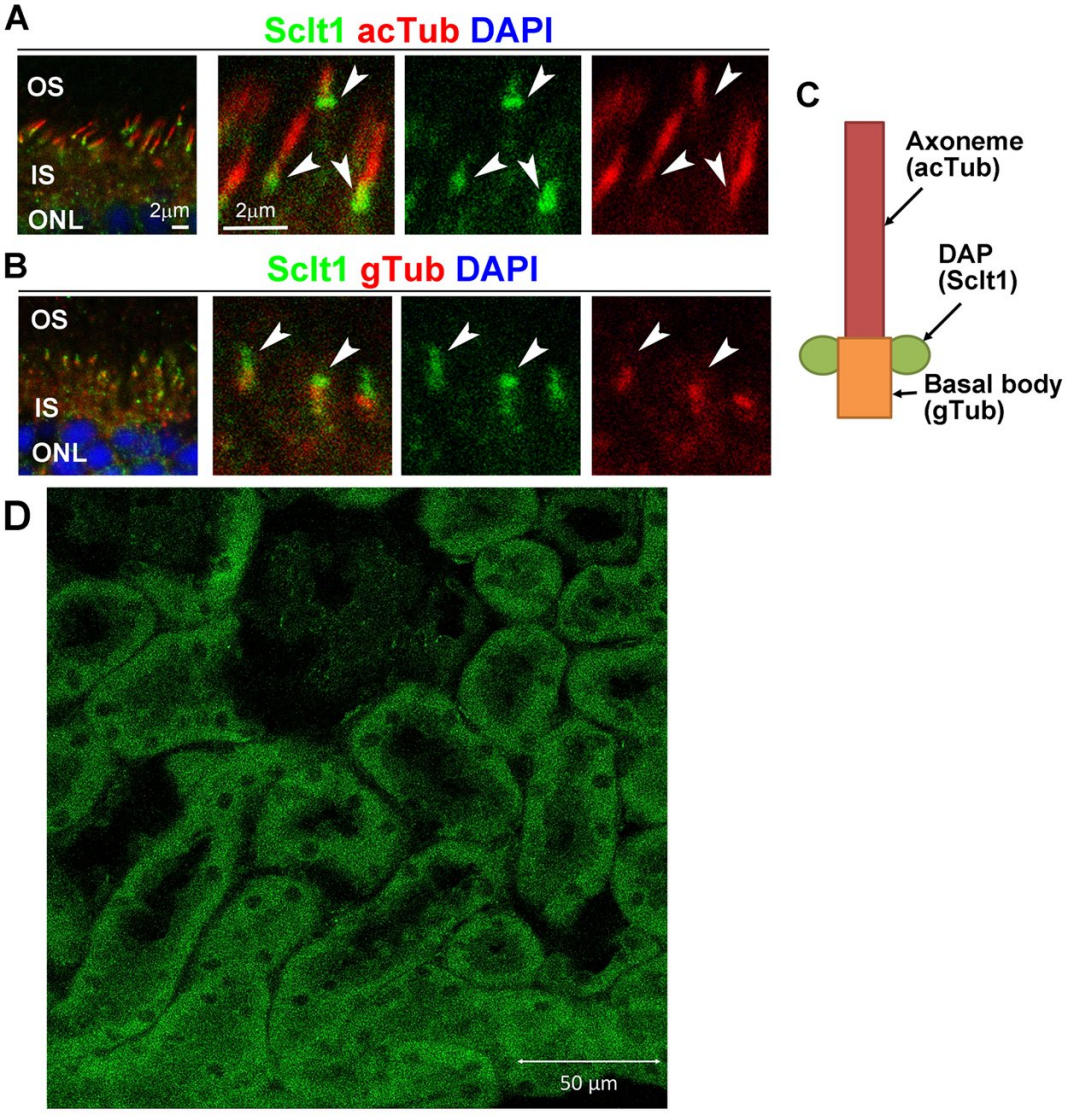
Localization of Sclt1 in the retinal photoreceptor cells and the kidney. (**A,B**) Retinal sections from the wild-type mice at P21 are immunostained with antibodies against Sclt1 (green in A and B, arrowheads), acetylated α-tubulin (a marker of the ciliary axoneme, red in A), and γ-tubulin antibodies (a maker for the basal body, red in B). Nuclei were stained with DAPI (blue). The Sclt1 signal is observed at the basal part of the photoreceptor ciliary axoneme and the distal part of the basal body. OS = outer segment, IS = inner segment, and ONL = outer nuclear layer. (**C**) Schematic representation of the photoreceptor connecting cilium, basal body and distal appendage (DAP). Sclt1 localizes to the DAP. (**D**) Sclt1 protein expression was observed in the tubule cells of the adult mouse kidney.

In the first step, we double-stained P21 mouse retinas using antibodies against Sclt1 and acetylated α-tubulin (a marker for the ciliary axoneme). The results indicated localization of Sclt1 at the base of the photoreceptor connecting the cilia (Fig. 7A,B, arrowheads), thereby indicating that Sclt1 is a ciliary protein. We then double-stained the P21 mouse retinas using antibodies against Sclt1 and γ-tubulin (a marker for the basal body), and found that Sclt1 is localized at the distal part of the photoreceptor basal bodies (Fig. 7C, arrowheads). In the adult kidney, Sclt1 protein expression was observed in the tubule cells (Fig. 7D).

## Discussion

In this study, using whole-exome sequencing, we identified compound heterozygous spice site variants in the *SCLT1* gene as a candidate locus for SLS. Both *SCLT1* variants, located in the exon-intron boundaries, have previously been unreported as pathogenic variants. Our genetic and transcript analysis clarified that the c.1218 + 3insT variant led to the exon 14 skipping (p.V383_M406del) (Fig. 6C,D). The other variant (c.1631A > G) primarily led to the exon 17 skipping (p.D480EfsX11) as well as to minor amounts of two transcripts (6 bps deletion in the last part of exon 17 [p.V543_K544del] and exons 17 and 18 skipping [p.D480E, S481_K610del]) (Fig. 6E,F). There have been two cases with inherited disorders with eye involvement associated *SCLT1* variants reported to date^20,21^. Adly *et al*. report that the homozygous *SCLT1* variant (c.290 + 2 T > C), which led to exon 5 skipping resulting in a frameshift and an 83 amino acids-truncated protein (p.K79VfsX4) during their transcript analysis, and the phenotype were associated with the extremely serious phenotype that exhibited the clinical features of Oro-Facio-Digital syndrome type IX complicated with severe coloboma^20^.

In contrast, although de Castro-Miro *et al*. did not perform transcript analysis, they did report finding the compound heterozygous *SCLT1* variants (c.778-2 A > T and c.827 G > A, p.R276H), and showed that the phenotype was associated with non-syndromic RP^21^. These results regarding the genotype-phenotype correlation appear to indicate the presence of a gradient of severity between the previous cases^20,21^ and our current study. In other words, the entire dysfunction of the SCLT1 protein (p.K79VfsX4) can lead to the more severe phenotype of the Oro-Facio-Digital syndrome type IX, in which the subject dies by the age of three months^20^. However, in the other patient, the function of SCLT1 protein with p.R276H, which is likely to be a hypomorphic variant, in at least one allele was shown to lead to a milder phenotype of the non-syndromic RP^21^.

Our current case carried the compound heterozygous variants, which resulted in mainly two SCLT1 transcripts, 72-bp in-frame deletion and frameshift deletion, producing p.V383_M406del and p.D480EfsX11, respectively (Fig. 6), and exhibited the medium phenotypes of SLS as compared with the two previously detailed cases^20,21^. Because it is possible that the p.V543_K544del (lacking only 24 amino acids compared with the full-length) might function as part of SCLT1 protein. Although up until the present the functional domain and motif of the SCLT1 protein has been unclear, we were able to show that the different phenotypic manifestations could be explained by the different transcripts with *SCLT1* variants between the previous cases^20,21^ and our current case.

Early-onset severe retinal degeneration and NPHP are the main features of SLS^1–3^. Moreover, many other symptoms have also been reported in patients with this disease^22,23^. These multi-organ disorders are clinical features of ciliopathies^19,24^. In fact, all of the 10 known causative SLS genes (*NPHP1*^6,7^, *NPHP2*^8^, *NPHP3*^9^, *NPHP4*^10^, *NPHP5/IQCB1*^11^, *NPHP6/CEP290*^12,13^, *NPHP10/SDCCAG8*^14^, *NPHP13/WRD19*^15,16^, *NPHP15/CEP164*^17^ and *TRAF3IP1*^18^), variants of which are known to cause SLS, have been shown to have a strong relationship to cilia^11,14,25–28^. Therefore, SLS is classified as one of the ciliopathies.

Using cultured cells, the Sclt1 protein has been shown to localize to distal appendages (DAPs), which is essential for ciliogenesis^29–31^. In tissues, Sclt1 protein expression was observed in tubule cells of the mouse kidney (Fig. 7D). A recent study has revealed that *Sclt1* knock-out mice (*Sclt1*^−/−^) exhibit typical ciliopathy phenotypes, including cystic kidney, cleft palate and polydactyly^32^, which overlapped with Oro-Facio-Digital syndrome type IX^20^. The histological findings of the *Sclt1*^−/−^ kidney were similar to those of our patient’s nephrected kidney (Fig. 3). These findings demonstrate that SCLT1 dysfunction is tightly associated with the progressive cystic kidney disease both in mouse and human. However, any eye complication has never been mentioned in the *Sclt1*^−/−^ mice study^32^. To date, the precise localization of Sclt1 in the retinal tissues has yet to be analyzed. Our immunohistochemical analysis using mouse retina suggested that Sclt1 localizes to the DAP of the photoreceptor basal body (Fig. 7), indicating that Sclt1 plays a role in ciliogenesis and/or cilia function in the photoreceptors. This result was consistent with our OCT findings that showed photoreceptor EZ disruption and marked thinning of the outer retinal layers in the patient (Fig. 2C). Interestingly, genetic variants in the *NPHP15/CEP164* and *NPHP18/CEP83* genes, which also can encode the components of DAPs similar to the *SCLT1* gene, have been reported to be the causes of the NPHP-related ciliopathies^17,33^. Although there have only been a small number of cases reported, a gradient of disease severity has been reported in *CEP164* variants, such as null variants causing more severe phenotypes of Meckel syndrome and Joubert syndrome in contrast to the hypomorphic variants, which cause the milder phenotypes of NPHP and SLS^17^. Furthermore, regarding the genotype-phenotype correlation for the *CEP83* variants, homozygous deletion variants that lead to truncated protein cause more severe phenotypes with multi-organ disorders as compared to the biallelic variants with at least one hypomorphic variant^33^. Highly similar genotype-phenotype correlations were also observed for the *SCLT1* variants. These included a more severe phenotype of the Oro-Facio-Digital syndrome type IX^20^, the typical phenotype for SLS seen in our current study, and the milder phenotype of non-syndromic RP^21^. The gradient of severity, ranging from more severe forms to less severe forms, may be the characteristic feature of the NPHP-related ciliopathies with DAP related gene variants.

In conclusion, new compound heterozygous splice site *SCLT1* variants were identified as being the cause of SLS. This is the first report to show that biallelic *SCLT1* variants underlay the development of SLS. Our findings additionally highlight the broad spectrum of clinical phenotypes that can be associated with the biallelic *SCLT1* variants.

## Methods

The protocol used for this study was approved by the Institutional Review Board of the Jikei University School of Medicine (approval ID: 24–231 6997) and the National Hospital Organization Tokyo Medical Center (approval ID: R18-029). The protocol adhered to the tenets of the Declaration of Helsinki. The patient (JU0947), her brothers and her parents provided written informed consent before participating in this study.

### Clinical study

The patient and her parents also provided written informed consent for publication of identifying clinical information/images in an online open-access publication. The patient (JU0947, II-2) underwent a comprehensive ophthalmic examination including BCVA, slit lamp examination, funduscopy, visual-field testing using GP (Haag Streit, Bern, Switzerland), wide-field fundus photographs and fundus autofluorescence images (Optos 200Tx, Optos, Dunfermline, United Kingdom), and spectral domain OCT (Carl Zeiss Meditec AG, Dublin, CA, USA). Full-field electroretinography using a light-emitting diode with a built-in electrode (LE-4000, Tomey, Nagoya, Japan) was recorded in accordance with the protocols of the International Society for Clinical Electrophysiology of Vision^34^. The procedure and conditions used in this study have been previously reported^35,36^.

### Genetic analysis

We obtained venous blood samples from the patient (II-2) and her Japanese family that included her unaffected and non-consanguineous parents (I-1, I-2) and brothers (II-1, II-3) (Fig. 2A). The Gentra Puregene Blood Kit (Qiagen, Hilden, Germany) was used to extract genomic DNA from peripheral blood leukocytes. Whole-exome sequencing was performed on the proband and her parents using previously described methods^37^. Briefly, we initially focused only on variants, including non-synonymous variants, splice acceptor and donor site variants, and short insertions and deletions (INDELs). Subsequently, we then filtered the variants based on the criterion that the frequency of a variant would be less than 1% in the 1000 Genomes database (http://www.1000genomes.org), the Exome Aggregation Consortium database (http://exac.broadinstitute.org), the Human Genetic Variation Database (http://www.genome.med.kyoto-u.ac.jp/SnpDB/), and the Tohoku Medical Megabank Organization database (https://ijgvd.megabank.tohoku.ac.jp). The remaining variants were winnowed by excluding variants that were found in the in-house database of exome data from 7 people without ocular diseases. Finally, we screened the remaining variants by using patterns of inheritance (homozygosity, compound heterozygosity, or *de novo* variants). The identified *SCLT1* variants were then confirmed by performing Sanger sequencing in all of the family members. For the Sanger sequencing, two primer pairs were used; a forward primer, 5′-ATTTACAGGTTGCAAACACC-3′ and a reverse primer, 5′-TGCTGCAAACATGTCTATCTG-3′ for exon 14, and a forward primer, 5′-GGCTTGTCAGTGAACAAAGG-3′ and a reverse primer, 5′-GTATGCCTGCCAAGTTCTAC-3′ for exon 17. We used the nucleotide sequence of the *SCLT1* gene from the NCBI Reference Sequence (NM_144643.2).

### SCLT1 RNA analysis

Total RNA was extracted from white blood cells from the patient and her parents using the QIAamp RNA Blood Mini Kit (Qiagen). For the cDNA synthesis, reverse transcription was performed by the PrimeScript 1st strand cDNA Synthesis Kit (Takara Bio Inc., Shiga, Japan). To analyze the c.1218 + 3insT variant, RT-PCR was conducted using the following primer pair; SCLT1-E13F (5′-AGACAGTTTCTCGGTTTGTAC-3′) in exon 13 and SCLT1-E17R (5′-CTTTGTTCACTGACAAGCCC-3′) in exon 17. Electrophoresis was performed using 3% agarose gels. The gel-extracted RT-PCR products were cloned using the Mighty Cloning Reagent Set (Blunt End) kit (Takara Bio Inc.). Each clone was directly sequenced using the vector reverse M13 primer (5′-CAGGAAACAGCTATGAC-3′). For analysis of the c.1631A > G variant, RT-PCR was performed using the following primer pair; 5′-end HEX-labeled SCLT1-E14F (5′-GAACTTTCAGCCCTTCAAATG-3′) in exon 14 and SCLT1-E20R (5′-GCCTGACTTAGACGCCTTTG-3′) in exon 20 or SCLT1-E14F and 5′-end FAM-labeled SCLT1-E20R. The cDNA was amplified using either 35 cycles or 40 cycles. Subsequently, GeneScan analysis was then conducted using GeneMapper software (Applied Biosystems/Life Technologies, Foster City, CA) as previously described^38^.

### Animal care and immunohistochemistry

All procedures conformed to the ARVO statement for the Use of Animals in Ophthalmic and Vision Research. All of the procedures were approved by the Institutional Safety Committee on Recombinant DNA Experiments (approval ID 04220) and the Animal Experimental Committees of the Institute for Protein Research (approval ID 29-01-0) at Osaka University. Mice were housed in a temperature-controlled room at 22 °C with a 12-hour light/dark cycle. Fresh water and a rodent diet were available at all times. Mice were sacrificed with carbon dioxide, and all efforts were made to minimize suffering. Immunohistochemistry was performed as previously described with some modification^39^. The mouse eyeballs were embedded in OCT compound 4583 (Sakura Finetek USA Inc, CA, USA) without fixation, frozen on dry ice for 5 min, and sectioned using a MICROM HM560 cryostat (ThermoFisher Scientific, Germany). Similarly, the adult ICR mouse (10 weeks old) kidneys were also prepared for immunohistochemistry, which was approved by the Animal Experimental Committees (approval ID 2016-001) at The Jikei University. Frozen 20 μm sections on slides were dried for 30 min at room temperature, rehydrated in PBS for 5 min, incubated with blocking buffer (5% normal goat serum, and 0.1% Triton X-100 in PBS) for 30 min, and incubated with the primary antibodies in blocking buffer for 4 hours at room temperature. Slides were washed with PBS and incubated with the secondary antibodies in blocking buffer for 2 hours at room temperature. The specimens were observed under a laser confocal microscope (LSM700, Carl Zeiss). We used the following primary antibodies for immunostaining: rabbit polyclonal anti-SCLT1 (HPA036561, 1:100, Sigma-Aldrich Corporation, St. Louis, MO, USA), mouse monoclonal anti-acetylated α-tubulin (Sigma-Aldrich Corporation, 6-11B-1, 1:1000), and anti-γ-tubulin antibodies (Sigma, GTU-88, 1:300). We used Cy3-conjugated (1:500, Jackson ImmunoResearch Laboratories, Baltimore, PA, USA) and Alexa Fluor 488-conjugated secondary antibodies (1:500, Sigma-Aldrich Corporation).

## Electronic supplementary material

Supplementary Information
