# Supplementary material for: Compound heterozygous splice site variants in the SCLT1 gene highlight an additional candidate locus for Senior-Løken syndrome
Source: Sci Rep. 2018 Nov 13;8:16733. doi: 10.1038/s41598-018-35152-6 (PMC6233217; doi:10.1038/s41598-018-35152-6)
Supplement: Supplementary file 1 — Supplementary Information [file 41598_2018_35152_MOESM1_ESM.pdf]

Supplementary Information

Title

Compound heterozygous splice site variants in the *SCLT1* gene highlight an additional candidate locus for Senior-Løken syndrome

Authors

Satoshi Katagiri, M.D., Ph.D.[1], Takaaki Hayashi, M.D., Ph.D.[1,2], Kazutoshi Yoshitake, Ph.D.[3], Noriyuki Murai, Ph.D.[4], Zenichi Matsui, M.D.[5], Hiroyuki Kubo, M.D.[1, 6], Hiroyuki Satoh, M.D., Ph.D.[5], Senya Matsufuji, M.D., Ph.D.[4], Tsuyoshi Takamura, M.D.[7], Takashi Yokoo, M.D., Ph.D.[7], Yoshihiro Omori, Ph.D. [8], Takahisa Furukawa, M.D., Ph.D. [8], Takeshi Iwata, Ph.D,[3] Tadashi Nakano, M.D., Ph.D.[1]

[1] Department of Ophthalmology, The Jikei University School of Medicine, Tokyo, Japan

[2] Department of Ophthalmology, Katsushika Medical Center, The Jikei University School of Medicine, Tokyo, Japan

[3] National Institute of Sensory Organs, National Hospital Organization Tokyo Medical Center, Tokyo, Japan

[4] Department of Molecular Biology, The Jikei University School of Medicine, Tokyo, Japan

[5] Department of Urology and Kidney transplants, Tokyo Metropolitan Children's Medical Center, Tokyo, Japan

[6] Department of Ophthalmology, Kanagawa Rehabilitation Hospital, Kanagawa, Japan

[7] Division of Nephrology and Hypertension, Department of Internal Medicine, The Jikei University School of Medicine, Tokyo, Japan

[8] Laboratory for Molecular and Developmental Biology, Institute for Protein Research, Osaka University, Osaka, Japan

Supplementary Table S1. Information on variants remaining after the filtering steps

Chrom	Position	Ref	Alt	State	Gene Name	Accession number	Exon	Nucleotide change	Amino Acid change	SNP ID	*in silico* analysis			Frequency		
											Polyphen2_HVAR_score	SIFT_score	PROVEAN_score	HGVD	ExAC	ExAC East Asian
4	128942997	T	C	Ref/Alt	*SCLT1*	NM_144643	17	c.A1631G	p.D480EfsX11 (main transcript) p.D480EfsX11 and p.D480E, S481_K610del (minor transcripts)	rs762215370	not evaluated	not evaluated	not evaluated	0.00137174	0.00001683	0.0002
4	128952766	T	TA	Ref/Alt	*SCLT1*	NM_144643	14	c.1218+3insT	p.V383_M406del	not reported	not evaluated	not evaluated	not evaluated	0	0	0
7	74051803	G	A	Ref/Alt	*ELN*	NM_001278913	13	c.G745A	p.V249M	rs199709542	0.476 (Probably damaging)	0.044 (Damaging)	-0.44 (Neutral)	0.00229148	0.00006609	0.0005
7	74063327	G	A	Ref/Alt	*ELN*	NM_001278913	24	c.G1633A	p.A545T	rs781861907	0.122 (Benign)	0 (Damaging)	-0.75 (Neutral)	0.00184332	0.0001	0.0029
17	7107061	G	A	Alt/Alt	*ASGR2*	NM_080914	6	c.C545T	p.A182V	rs199839502	0.998 (Damaging)	0.001 (Damaging)	-3.85 (Damaging)	0.00722674	0.0000659	0.0009

Chrom = chromosome, Ref = reference allele, Alt = alternative allele, The position of variants is determined using the Human Genome reference (GRCh38/hg38), HGVD = human genetic variation database (http://www.hgvd.genome.med.kyoto-u.ac.jp/index.html), ExAC = Exome Aggregation Consortium database (http://exac.broadinstitute.org), Polyphen-2 (http://genetics.bwh.harvard.edu/pph2/), SIFT (http://sift.jcvi.org), PROVEAN (http://provean.jcvi.org), The transcripts of *SCLT1* variants were determined by RNA analysis.

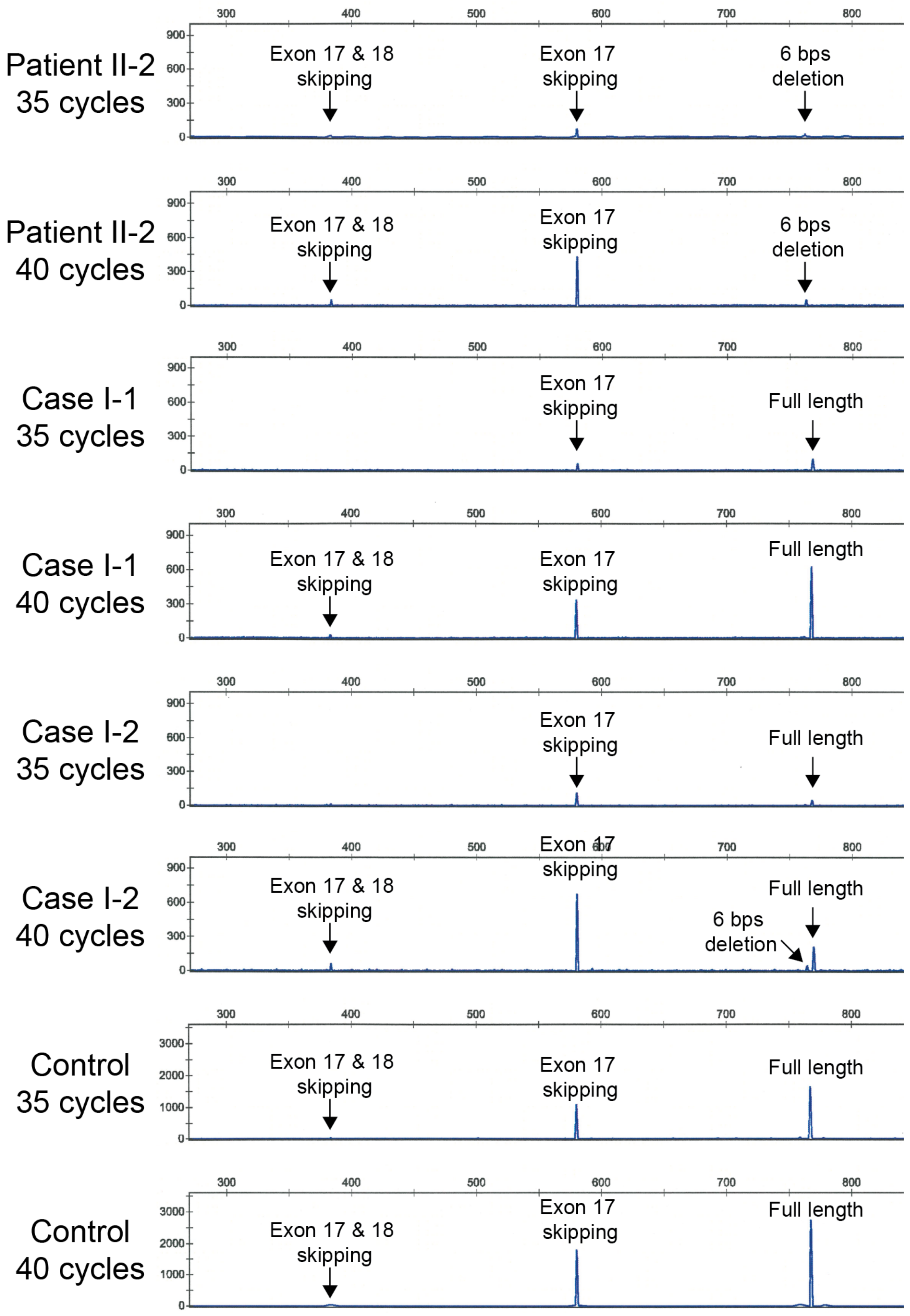

Supplemental Figure S1. SCLT1 transcript analysis

The results of GeneMapper using the reverse transcription-PCR product with a primer pair of SCLT1-E14F and FAM labeled SCLT1-E20R. Each sample shows different patterns and four distinct peaks, which are approximately 768, 763, 580, and 383 bps. The cDNA was amplified using either 35 cycles or 40 cycles. The dye signal intensity from 35 cycles is weaker than that from 40 cycles. Note that the full length of the SCLT1 mRNA transcript is not detected in Patient II-2.
